# Cassane diterpenoids with α-glucosidase inhibitory activity from the fruits of *Pterolobium macropterum*

**DOI:** 10.3762/bjoc.19.47

**Published:** 2023-05-11

**Authors:** Sarot Cheenpracha, Ratchanaporn Chokchaisiri, Lucksagoon Ganranoo, Sareeya Bureekaew, Thunwadee Limtharakul, Surat Laphookhieo

**Affiliations:** 1 Division of Chemistry, School of Science, University of Phayao, Phayao 56000, Thailand; 2 Department of Energy Science and Engineering, Vidyasirimedhi Institute of Science and Technology (VISTEC), Wangchan, Rayong 21210, Thailand; 3 Department of Chemistry and Center of Excellence for Innovation in Chemistry, Faculty of Science, the Graduate School and Research Center on Chemistry for Development of Health Promoting Products from Northern Resources, Chiang Mai University, Chiang Mai 50200, Thailand; 4 Center of Chemical Innovation for Sustainability (CIS) and School of Science, Mae Fah Luang University, Chiang Rai 57100, Thailand; 5 Medicinal Plants Innovation Center of Mae Fah Luang University, Chiang Rai 57100, Thailand

**Keywords:** α-glucosidase inhibitory activity, cassane diterpenoid, Fabaceae, medicinal plant, *Pterolobium macropterum*

## Abstract

Two new cassane diterpenoids, 14β-hydroxycassa-11(12),13(15)-dien-12,16-olide (**1**) and 6′-acetoxypterolobirin B (**3**), together with a known analogue, identified as 12α,14β-dihydroxycassa-13(15)-en-12,16-olide (**2**), were isolated from the fruits of *Pterolobium macropterum*. Compound **1** is a cassane diterpenoid with a Δ^11(12)^ double bond conjugated with an α,β-butenolide-type, whereas compound **3** is a dimeric caged cassane diterpenoid with unique 6/6/6/6/6/5/6/6/6 nonacyclic ring system. The structures of **1** and **3** were characterized by extensive spectroscopic analysis combined with computational ECD analyses. The α-glucosidase inhibitory activity of isolated compounds was evaluated, and compounds **1** and **3** showed significant α-glucosidase inhibitory activity with IC_50_ values of 66 and 44 μM.

## Introduction

Diabetes mellitus is a common metabolic disease that affects how the body uses blood glucose. In 2021, 537 million patients suffered from diabetes worldwide, and the number is feared to increase to 783 million in 2045 [1]. Type 2 diabetes account for the majority of the cases [2]. Currently, inhibition of α-glucosidase, the enzyme responsible for the hydrolysis of carbohydrates in the body, is widely used for the management of type 2 diabetes. The agents, such as acarbose, miglitol, and voglibose, can retard the digestion and absorption of dietary carbohydrates [3–4]. Some cassane-type diterpenoids such as pulcherrimin C and 6β-cinnamoyl-7β-hydroxyvouacapen-5α-ol, have been reported to exhibit significant α-glucosidase inhibitory activity [5].

The genus *Pterolobium*, comprising approximately 10 species distributed widely in Africa, China, and Thailand [6], is flowering shrubs belonging to Fabaceae. There are only four species known in Thailand [7], and some of them have been applied as antihemorrhoid [8]. Some species of this genus have revealed cassane diterpenoids as mainly secondary metabolites, which have shown interesting biological activities such as cytotoxicity and anti-inflammatory activity [9–11].

**Figure 1:**
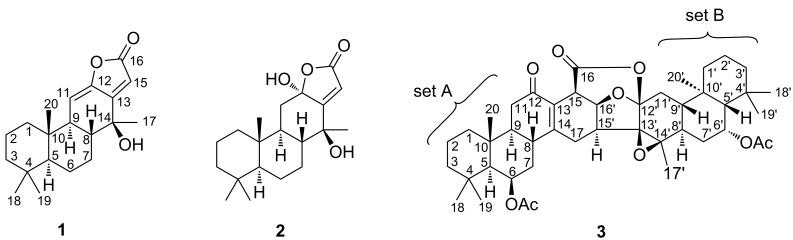
Chemical structures of **1**-**3** isolated from *P. macropterum*.

*Pterolobium macropterum* Kurz is a woody climbing shrub that is mainly distributed in the northern Thailand. Its roots are used in the medicinal plant therapy to treat toothache, fever, and to promote wound healing [11]. Previous phytochemical investigations of *P. macropterum* have revealed that this plant is a source of cassane diterpenoids featuring the structure of three cyclohexane rings with a constructed furan ring or an α,β-butenolide ring [9–10]. Recently, only two caged cassane diterpenoid dimers isolated from the fruits of this plant have been reported [11]. Some cassane-type diterpenes displayed to exhibit diverse biological properties, including anti-inflammatory [12], antimalarial [13], antitumor [14], antiviral [15], antibacterial [16], antiproliferative [13], and immunomodulatory [17] activities. As part of our studies on Thai medicinal plants, an investigation of the fruits of *P. macropterum* resulted in the isolation of one new cassane diterpenoid, 14β-hydroxycassa-11(12),13(15)-dien-12,16-olide (**1**), one new caged cassane diterpenoid dimer featuring a 6/6/6/6/6/5/6/6/6 nonacyclic ring system, 6′-acetoxypterolobirin B (**3**), and one known compound (Figure 1).

## Results and Discussion

The fruits of *P. macropterum* were extracted using MeOH to give a crude extract. After removal of the organic solvent, the extract was separated by repeated silica gel column chromatography as well as by Sephadex LH-20 to afford two new and one known cassane diterpenoids, identified as 12α,14β-dihydroxycassa-13(15)-en-12,16-olide (**2**) [18].

Compound **1** was isolated as an amorphous white powder. The molecular formula was determined as C_20_H_28_O_3_ from the HRESI–TOF–MS analysis with a [M + H]^+^ ion peak at *m*/*z* 317.2107 (calcd C_20_H_29_O_3_, 317.2111) and was considered to have 7 degrees of unsaturation. Its IR absorptions revealed the presence of hydroxy (3429 cm^−1^) and carbonyl (1733 cm^−1^) groups. The UV absorption band maximum at λ_max_ 283 nm and five downfield-shifted carbon signals at δ_C_ 169.8 (C-16), 163.8 (C-13), 149.3 (C-12), 111.6 (C-11), and 109.6 (C-15) in the ^13^C NMR data suggested the presence of the α,β-butenolide ring conjugated with one extra double bond [12]. In the ^1^H NMR spectrum (Table 1), the resonances for four methyls [δ_H_ 1.30 (3H, s, H_3_-17), 0.90 (3H, s, H_3_-18), 0.85 (3H, s, H_3_-20), and 0.83 (3H, s, H_3_-19)], two olefinic protons [δ_H_ 6.03 (1H, br s, H-15) and 5.86 (1H, br s, H-11)] were observed. The ^13^C NMR and DEPT spectra, combined with HMQC correlations (Table 1) showed 20 resonances for carbon signals accounting for four methyls, five sp^3^ methylenes, five methines (two olefinics at δ_C_ 111.6, 109.6), and six quaternary carbons (one carbonyl at δ_C_ 169.8, two olefinics at δ_C_ 163.8, 149.3, and one oxygenated sp^3^ at δ_C_ 72.2). The ^1^H and ^13^C NMR spectroscopic data of **1** showed great similarity to those of 12α,14β-dihydroxycassa-13(15)-en-12,16-olide (**2**) isolated from *Caesalpinia bonduc* [18]*.* The difference evident was that compound **1** displayed an extended conjugate π-system with an α,β-unsaturated γ-lactone ring.

**Table 1:** ^1^H (500 MHz) and ^13^C NMR (125 MHz) data for compounds **1** and **3**.

position	**1** ^a^			**3** ^a^	
		
	δ_C_	δ_H_, mult (*J* in Hz)		δ_C_	δ_H_, mult (*J* in Hz)

1	38.5, CH_2_	1.85 m; 1.06 m		40.6, CH_2_	1.69 m; 1.61 m
2	18.6, CH_2_	1.64 m; 1.53 m		18.7, CH_2_	1.64 m; 1.47 m
3	41.8, CH_2_	1.45 m; 1.22 m		43.8, CH_2_	1.40 m; 1.15 m
4	33.4, C			33.8, C	
5	54.8, CH	0.99 dd (11.7, 2.7)		55.2, CH	1.03 m
6	21.1, CH_2_	1.80 m; 1.44 m		68.8, CH	5.53 br q (2.7)
7	25.1, CH_2_	2.06 m		37.6, CH_2_	2.72 m
8	46.9, CH	1.84 br t (11.0)		35.3, CH	2.28 m
9	52.2, CH	1.88 br d (11.0)		53.6, CH	1.56 m
10	37.8, C			37.6, C	
11	111.6, CH	5.86 br s		37.7, CH_2_	2.60 dd (14.7, 2.9) 2.37 t (14.7)
12	149.3, C			197.9, C	
13	163.8, C			128.0, C	
14	72.2, C			155.4, C	
15	109.6, CH	6.03 br s		37.0, CH	4.59 dd (6.6, 1.9)
16	169.8, C			167.0, C	
17	22.3, CH_3_	1.30 s		25.3, CH_2_	2.54 dd (20.0, 10) 2.20 m
18	33.2, CH_3_	0.90 s		33.2, CH_3_	0.95 s
19	21.5, CH_3_	0.83 s		23.3, CH_3_	1.00 s
20	15.5, CH_3_	0.85 s		16.7, CH_3_	1.20 s
1′				40.7, CH_2_	1.69 m; 1.61 m
2′				18.7, CH_2_	1.64 m; 1.47 m
3′				43.8, CH_2_	1.40 m; 1.15 m
4′				33.8, C	
5′				55.1, CH	1.03 m
6′				69.4, CH	5.56 br q (2.7)
7′				35.5, CH_2_	2.29 m
8′				36.0, CH	2.73 m
9′				43.1, CH	1.40 m
10′				36.8, C	
11′				30.9, CH_2_	2.18 m
12′				104.1, C	
13′				71.3, C	
14′				65.4, C	
15′				33.8, CH	2.73 m
16′				73.7, CH	4.55 t (6.6)
17′				16.2, CH_3_	1.23 s
18′				33.1, CH_3_	0.95 s
19′				23.2, CH_3_	0.98 s
20′				16.3, CH_3_	1.09 s
6-OCO*CH*_3_				21.8, CH_3_	2.06 s
6-O*CO*CH_3_				170.7, C	
6′-OCO*CH*_3_				21.9, CH_3_	2.10 s
6′-O*CO*CH_3_				170.4, C	

^a^NMR data were recorded in chloroform-*d.*

**Figure 2:**
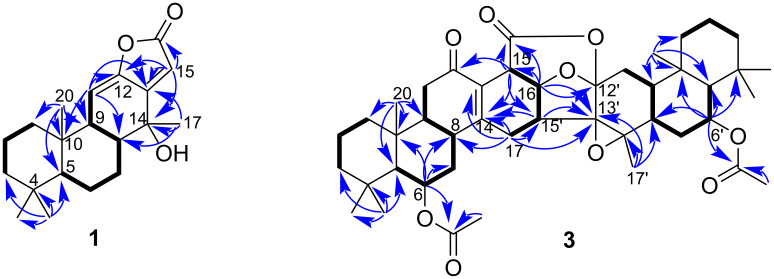
Key ^1^H,^1^H-COSY, and HMBC correlations of **1** and **3**.

The HMBC cross-peaks (Figure 2) from H-11 (δ_H_ 5.86) to C-10, C-12, and C-13, and from H-15 (δ_H_ 6.03) to C-12, C-13 and C-14 allowed the location of an extended conjugated π-system at C-11 and C-12. Moreover, the downfield shift of C-14 (δ_C_ 72.2) and the HMBC correlation between H_3_-17 and C-14 as well as the appearance of the H_3_-17 as a singlet signal confirmed the connection of a hydroxy group at C-14.

**Figure 3:**
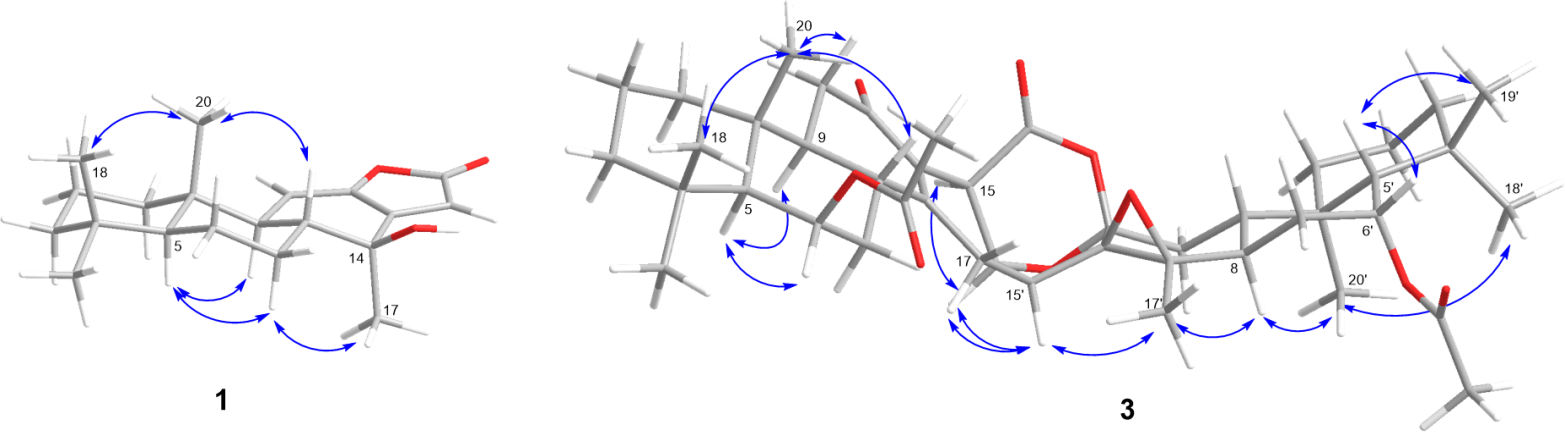
Selected NOESY cross peaks of **1** and **3**.

The relative configuration of **1** was characterized by NOESY spectra. In the NOESY experiment (Figure 3), the cross-peak between H-8 and H_3_-20 suggested these protons to be *syn* oriented. In addition, the cross-peaks between H-5/H-9, H-5/H-7α and H-7α/H_3_-17 suggested H_3_-17 to be α-oriented.

**Figure 4:**
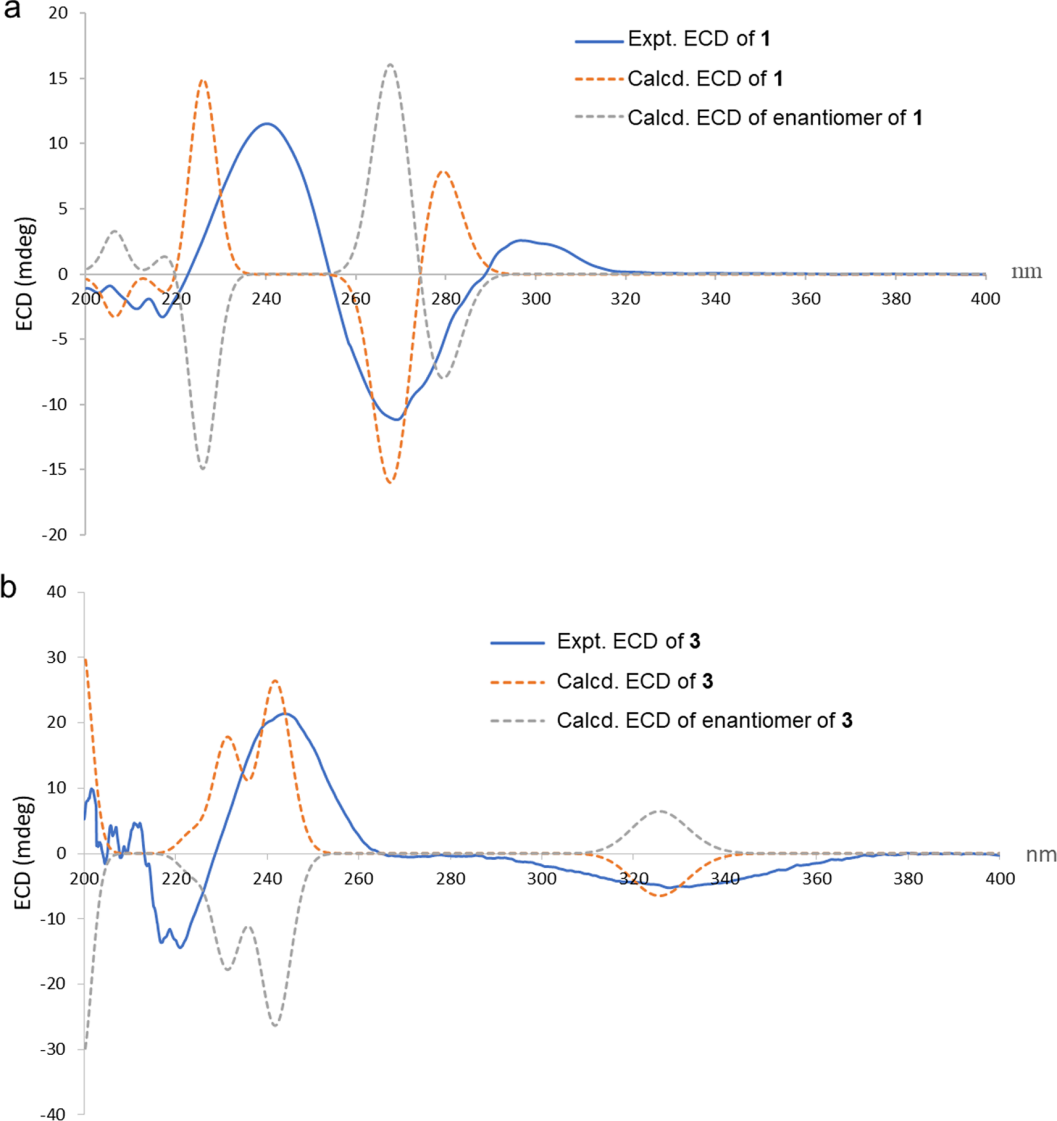
Measured and predicted ECD spectra of **1** and **3**.

Comparison of the specific rotation was used to establish the absolute configuration of **1**. The specific rotation of **1** (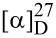 −22 (*c* 0.01, MeOH)) was similar to the reported data of **2** (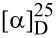 −36 (*c* 0.01, MeOH); lit. 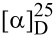 −44 (*c* 0.05, MeOH)) [18], confirming the same absolute configuration these compounds should be derived from the same biosynthetic pathway. In addition, the ECD spectra of (5*S*,8*R*,9*S*,10*R*,14*S*)-**1** and its enantiomer were calculated at the B3LYP functional using a TD–DFT method [19]. As illustrated in Figure 4a, the measured ECD curve was compared to the predicted ECD curve of (5*S*,8*R*,9*S*,10*R*,14*S*)-**1**, indicating that the measured and predicted ECD spectra were similar except for a blue-shift in the ECD spectrum. Thus, the structure of **1** was characterized as shown.

Compound **3** was obtained as a colorless oil. The molecular formula was assigned to be C_44_H_60_O_9_ based on the HRESI–TOF–MS analysis with a [M + H]^+^ ion peak at *m/z* 733.4305 (calcd for C_44_H_61_O_9_, 733.4310) and NMR data, implying 15 degrees of unsaturation. The IR absorption band at 1724 cm^−1^ suggested the presence of α,β-unsaturated γ-lactone functionality. The ^13^C NMR and DEPT spectra in combination with HMQC data (Table 1) showed resonances of 44 carbons which were classified as nine methyls, 11 methylenes, 11 methines (three oxygenated sp^3^ at δ_C_ 73.7, 69.4, 68.8), and 13 quaternary carbons (four carbonyls at δ_C_ 197.9, 170.7, 170.4, 167.0, two olefinics at δ_C_ 155.4, 128.0, and three oxygenated at δ_C_ 104.1, 71.3, 65.4). Assuming a cassane-type diterpene skeleton, the ^1^H and ^13^C NMR spectra (Table 1) displayed two sets (A and B) of the characteristic signals of seven methyl singlets [set A: δ_H_/δ_C_ 1.20 (s, H_3_-20)/16.7, 1.00 (s, H_3_-19)/23.3, 0.95 (s, H_3_-18)/33.2; set B: δ_H_/δ_C_1.23 (s, H_3_-17′)/16.2, 1.09 (s, H_3_-20′)/16.3, 0.98 (s, H_3_-19′)/23.2, 0.95 (s, H_3_-18′)/33.1], three oxygenated methines [set A : δ_H_/δ_C_ 5.53 (br q, *J* = 2.7 Hz, H-6)/68.8; set B : δ_H_/δ_C_ 5.56 (br q, *J* = 2.7 Hz, H-6′)/69.4, 4.55 (t, *J* = 6.6 Hz, H-16′)/73.7], and two acetoxy groups [set A: δ_H_ 2.06 (s, 6-OCOCH_3_)/δ_C_ 21.8, and 170.7; set B: δ_H_ 2.10 (s, 6′-OCOCH_3_)/δ_C_ 21.9 and 170.4]. Careful analysis of the NMR data indicated the presence of a dimeric cassane-type diterpene skeleton whose NMR spectra resembled those of pterolobirin B [11], an unprecedented caged cassane diterpenoid dimer with unique 6/6/6/6/6/5/6/6/6 nonacyclic ring system. The minor difference was the additional acetoxy group (δ_H_ 2.10) at C-6′. This conclusion was suggested by the HMBC correlations (Figure 2) from H-6′ to 6′-O*CO*CH_3_ (δ_C_ 170.4) and C-10′ (δ_C_ 36.8), and from H_3_-20′ to C-5′ (δ_C_ 55.1) and C-10′, combined with the spin system CH(5′)-CH(6′)-CH_2_(7′)-CH(8′)-CH(9′)-CH_2_(11′) found in the COSY spectrum (Figure 2). The COSY correlations between H-15/H-16′, H-16′/H-15′ and H-15′/H_2_-17, and the HMBC cross-peaks from H-15′ to C-14 (δ_C_ 155.4), C-15 (δ_C_ 37.0), C-12′ (δ_C_ 104.1), and C-14′ (δ_C_ 65.4), and from H-16′ to C-13 (δ_C_ 128.0), C-16 (δ_C_ 167.0), C-17 (δ_C_ 25.3), C-12′, and C-13′ (δ_C_ 71.3) clearly indicated the two C–C bond linkages of both units through the C-15/C-16′ and C-17/C-15′ bonds. Furthermore, the aforementioned ring structure and functionalities accounting for 13 out of 15 degrees of unsaturation required the presence of two heterocyclic rings in the molecule. The presence of an ester carbonyl signal (δ_C_ 167.0) and a deshielded oxygenated carbon resonance at C-12′ (δ_C_ 104.1) implied the formation of six-membered ring via an ester bond between C-16 and C-12′. In addition, an epoxide moiety at C-13′ and C-14′ was further supported by the downfield shift of C-13′ (δ_C_ 71.1) and C-14′ (δ_C_ 65.4) and the cross-peaks from H_3_-17′/H-15′ to C-13′ and C-14′ in the HMBC spectrum.

In the NOESY experiments of **3** (Figure 3), the interactions of H_3_-20 to H_3_-18 and H-8 indicated the β-orientations, while the cross-peaks of H_3_-20′ to H_3_-18′ and H-8′ revealed the α-orientations of these protons. The protons H-6 and H-6′ are α- and β-oriented, respectively, as indicated by NOESY cross peaks from H-5 to H-6 and H-9; and from H-5′ to H-6′ and H_3_-19′, combined with the small coupling constants of H-6 (*J* = 2.7 Hz) and H-6′ (*J* = 2.7 Hz). The *syn* orientations of H-15, H-15′, H-16′ and H_3_-17′ were established from the NOESY correlations of H-15′ to H_3_-17′, H-16′ and of H-15 to H-16′. From above information, the relative configuration of C-12′ was assigned and supported by the biosynthetic pathway based on a Diels–Alder adduct, thus displaying the same relative configuration found in pterolobirin B [11]. The absolute configuration of **3** was thus elucidated as 5*S*,6*R*,8*R*,9*S*,10*R*,15*R*,5′*S*,6′*R*,8′*R*,9′*S*,10′*R*,12′*S*,13′*R*,14′*R*,15′*S*,16′*S* and the measured ECD spectrum (Figure 4b) with the positive at 243 nm and negative at 330 nm CEs, is very well matched with the ECD curve of pterolobirin B [11]. Although, the predicted ECD data is not in good agreement with the measured ECD data. It is noted that the calculation could not completely simulate the experimental results depend on the level of theory and basis set as well as the polarity of solvent. Finally, comparison of the specific rotation was used to establish the absolute configuration. Pterolobirin B showed 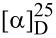 −72 (*c* 0.1, CHCl_3_) [11] and **3** showed 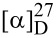 −87 (*c* 0.01, MeOH), which also supports the absolute configuration. Thus, the structure of **3** was assigned as shown.

The isolated compounds were evaluated for their α-glucosidase inhibitory activity [20]. Compounds **1** and **3** exhibited significant α-glucosidase inhibitory activity with IC_50_ values of 66 and 44 µM, respectively, which showed stronger inhibitory activity than the positive control, acarbose (IC_50_ 178 μM). Compound **2** was inactive in this assay, with IC_50_ value >200 μM, which suggested that a Δ^11(12)^ double bond might be important for the α-glucosidase inhibitory activity.

## Conclusion

In conclusion, two new cassane diterpenoids, 14β-hydroxycassa-11(12),13(15)-dien-12,16-olide (**1**) and 6′-acetoxypterolobirin B (**3**) together with one known analogue were isolated from the MeOH extract of *P. macropterum* fruits. Their structures and absolute configurations of **1** and **3** were established by spectroscopic analyses and ECD data. Compound **1** has an extended conjugated π-system with an α,β-unsaturated γ-lactone ring at the Δ^11(12)^ double bond, while compound **3** is caged cassane diterpenoid dimers with unique 6/6/6/6/6/5/6/6/6 nonacyclic ring system. Only two caged cassane diterpenoid dimers with unique 6/6/6/6/6/5/6/6/6 nonacyclic ring system were isolated previously from the same plant [11]. Biological evaluation revealed that compounds **1** and **3** exhibited significant α-glucosidase inhibitory activity with IC_50_ values of 66 and 44 μM, respectively.

## Experimental

### General experimental procedures

Optical rotations were measured on a JASCO P-2000 polarimeter in MeOH. The UV spectra were recorded on a PerkinElmer UV–vis spectrophotometer. ECD spectra were acquired on a JASCO J-1500 circular dichroism spectrometer. FTIR spectra were obtained using a PerkinElmer FTS FT-IR spectrophotometer. NMR spectra were obtained on a Bruker NEO 500 MHz NMR Ultra Shield. Chemical shifts are referenced in parts per million (δ) in the deuterated solvents (CDCl_3_) using TMS as an internal standard. An Agilent 1290 infinity II/Q-TOFMS mass spectrometer was employed to acquire HRESI–TOF–MS spectra. Column chromatography (CC) was carried out on silica gel 60 (70–230 mesh, Merck, Darmstadt, Germany), and Sephadex LH-20 (GE Healthcare). Thin-layer chromatography (TLC) was performed on silica gel 60 F_254_ plates (Merck, Darmstadt, Germany) using precoated aluminum plates for analytical purposes.

### Plant material

Fresh fruits of *Pterolobium macropterum* Kurz were collected from Song Khwae District, Nan Province, Thailand (GPS: 19°16'51.5"N 100°43'30.3"E) in August 2021 and identified by Mr. Martin van de Bult, Doi Tung Development Project. A voucher specimen (UP-CNP003) was deposited at the Chemistry of Natural Products for Sustainability Laboratory, School of Science, University of Phayao.

### Extraction and isolation

The fresh fruits of *P. macropterum* (0.2 kg) were ground and soaked with MeOH (3 × 2 L) at room temperature for 3 days. The solvent was evaporated under reduced pressure at 40 °C, affording MeOH extract (10.5 g). The extract was subjected to silica gel column chromatography (CC) (70–230 mesh, 2 × 60 cm) eluting with hexanes–acetone (100:0 → 0:100, v/v) to afford 10 fractions (Fr.1–Fr.10). Fr.5 (142.0 mg) was further fractionated over a Sephadex LH-20 column with a mixture of CH_2_Cl_2_–MeOH (1:1, v/v) affording five subfractions (Fr.5.1–Fr5.5). Subfraction Fr.5.2 (17.2 mg) was chromatographed over silica gel CC eluting with acetone–hexanes (1:19, v/v) to give compound **3** (1.1 mg). Fr.6 (842.0 mg) was chromatographed over silica gel CC eluting with acetone–hexanes (1:19, v/v), and then purified by silica gel CC using 100% CH_2_Cl_2_ to afford compound **1** (12.5 mg). Chromatographic purification of Fr.8 (1.2 g) over a Sephadex LH-20 column with a mixture of CH_2_Cl_2_–MeOH (1:1, v/v) afforded three subfractions (Fr.8.1–Fr8.3). Fr.8.2 (257.2 mg) was purified by silica gel CC eluting with acetone–hexanes (1:9, v/v), to provide five subfractions (Fr.8.2.1–Fr.8.2.5). Subfraction Fr.8.2.5 (28.8 mg), was chromatographed over silica gel CC eluting with 100% CH_2_Cl_2_ afforded compound **2** (6.9 mg).

14β-Hydroxycassa-11(12),13(15)-dien-12,16-olide (**1**): amorphous, white powder; 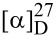 −22 (*c* 0.01, MeOH); UV (MeOH) λ_max_ (log ε) 283 (4.20) nm; ECD (0.001, MeOH) λ_max_ (Δε) 241 (+11.5), 271 (−10.4), 297 (+2.5) nm; IR (neat) ν_max_ 3429, 2926, 1733, 1664, 1603, 1263 cm^−1^; ^1^H NMR (500 MHz, CDCl_3_) and ^13^C NMR (125 MHz, CDCl_3_) spectra, see Table 1; HRESI–TOF–MS *m/z*: 317.2107 [M + H]^+^ (calcd for C_20_H_29_O_3_, 317.2111).

6′-Acetoxypterolobirin B (**3**): colorless oil; 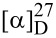 −87 (c 0.01, MeOH); UV (MeOH) λ_max_ (log ε) 243 (3.63) nm; ECD (0.001, MeOH) λ_max_ (Δε) 243 (+21.1), 330 (−4.70) nm; IR (neat) *ν*_max_: 2973, 1724, 1660, 1508, 1733 cm^−1^; ^1^H NMR (500 MHz, CDCl_3_) and ^13^C NMR (125 MHz, CDCl_3_) spectra, see Table 1; HRESI–TOF–MS *m/z*: 733.4305 [M + H]^+^ (calcd for C_44_H_61_O_9_, 733.4310).

### α-Glucosidase inhibitory assay

α-Glucosidase inhibitory activity was performed according to experimental literature with slight modification [20]. α-Glucosidase (0.05 U/mL) and substrate, *p*-nitrophenyl-α-ᴅ-glucopyronoside (p-NPG) (1 mM) were dissolved in 0.1 M sodium phosphate buffer (pH 6.9). Fifty μL of sample (1 mg/mL in 10% DMSO) and 50 μL of α-glucosidase were preincubated at 37 °C for 10 min in a 96 well plate. The substrate solution (50 μL) was added to the mixture to start the reaction, with further incubation at 37 °C for 20 min. The reaction was terminated by adding 1 mL of 0.3 M Na_2_CO_3_. Enzymatic activity was quantified by measuring the absorbance at 405 nm. The percent inhibition of activity was calculated as (A_0_ − A_1_)/A_0_ × 100, where A_0_ is the absorbance of control, and A_1_ is the absorbance with the sample. Acarbose was used as a standard drug and all experiments were evaluated in triplicate.

## Supporting Information

File 1 Copies of NMR spectra for compounds **1** and **3**.
