# Supplementary material for: Cassane diterpenoids with α-glucosidase inhibitory activity from the fruits of Pterolobium macropterum
Source: Beilstein J Org Chem. 2023 May 11;19:658–65. doi: 10.3762/bjoc.19.47 (PMC10186258; doi:10.3762/bjoc.19.47)
Supplement: File 1 — Copies of NMR spectra for compounds 1 and 3. [file Beilstein_J_Org_Chem-19-658-s001.pdf]

BEILSTEIN JOURNAL OF ORGANIC CHEMISTRY

# Supporting Information

for

# Cassane diterpenoids with α-glucosidase inhibitory activity from the fruits of *Pterolobium macropterum*

Sarot Cheenpracha, Ratchanaporn Chokchaisiri, Lucksagoon Ganranoo, Sareeya Bureekaew, Thunwadee Limtharakul and Surat Laphookhieo

*Beilstein J. Org. Chem.* **2023,** *19,* 658–665. doi:10.3762/bjoc.19.47

## Copies of NMR spectra for compounds 1 and 3

License and Terms: This is a supporting information file under the terms of the Creative Commons Attribution License (https://creativecommons.org/licenses/by/4.0). Please note that the reuse, redistribution and reproduction in particular requires that the author(s) and source are credited and that individual graphics may be subject to special legal provisions.
The license is subject to the *Beilstein Journal of Organic Chemistry* terms and conditions: (https://www.beilstein-journals.org/bjoc/terms)

**Table of contents**


| | Page |
|---|---|
| **Figure S1**. $^{1}H$ NMR (500 MHz, $CDCl_3$) spectrum of 14β-hydroxycassa-11(12),13(15)-dien-12,16-olide (**1**) | S3 |
| **Figure S2**. $^{13}C$ NMR (125 MHz, $CDCl_3$) spectrum of 14β-hydroxycassa-11(12),13(15)-dien-12,16-olide (**1**) | S3 |
| **Figure S3**. DEPT135 spectrum of 14β-hydroxycassa-11(12),13(15)-dien-12,16-olide (**1**) | S4 |
| **Figure S4**. $^{1}H$-$^{1}H$ COSY spectrum of 14β-hydroxycassa-11(12),13(15)-dien-12,16-olide (**1**) | S4 |
| **Figure S5**. HMQC spectrum of 14β-hydroxycassa-11(12),13(15)-dien-12,16-olide (**1**) | S5 |
| **Figure S6**. HMBC spectrum of 14β-hydroxycassa-11(12),13(15)-dien-12,16-olide (**1**) | S6 |
| **Figure S7**. NOESY spectrum of 14β-hydroxycassa-11(12),13(15)-dien-12,16-olide (**1**) | S7 |
| **Figure S8**. $^{1}H$ NMR (500 MHz, $CDCl_3$) spectrum of 6′-acetoxypterolobirin B (**3**) | S8 |
| **Figure S9**. $^{13}C$ NMR (125 MHz, $CDCl_3$) spectrum of 6′-acetoxypterolobirin B (**3**) | S8 |
| **Figure S10**. DEPT135 spectrum of 6′-acetoxypterolobirin B (**3**) | S9 |
| **Figure S11**. $^{1}H$,$^{1}H$ COSY spectrum of 6′-acetoxypterolobirin B (**3**) | S9 |
| **Figure S12**. HMQC spectrum of 6′-acetoxypterolobirin B (**3**) | S10 |
| **Figure S13**. HMBC spectrum of 6′-acetoxypterolobirin B (**3**) | S11 |
| **Figure S14**. NOESY spectrum of 6′-acetoxypterolobirin B (**3**) | S11 |
| **Figure S15**. Magnified NOESY spectrum of 6′-acetoxypterolobirin B (**3**) | S13 |

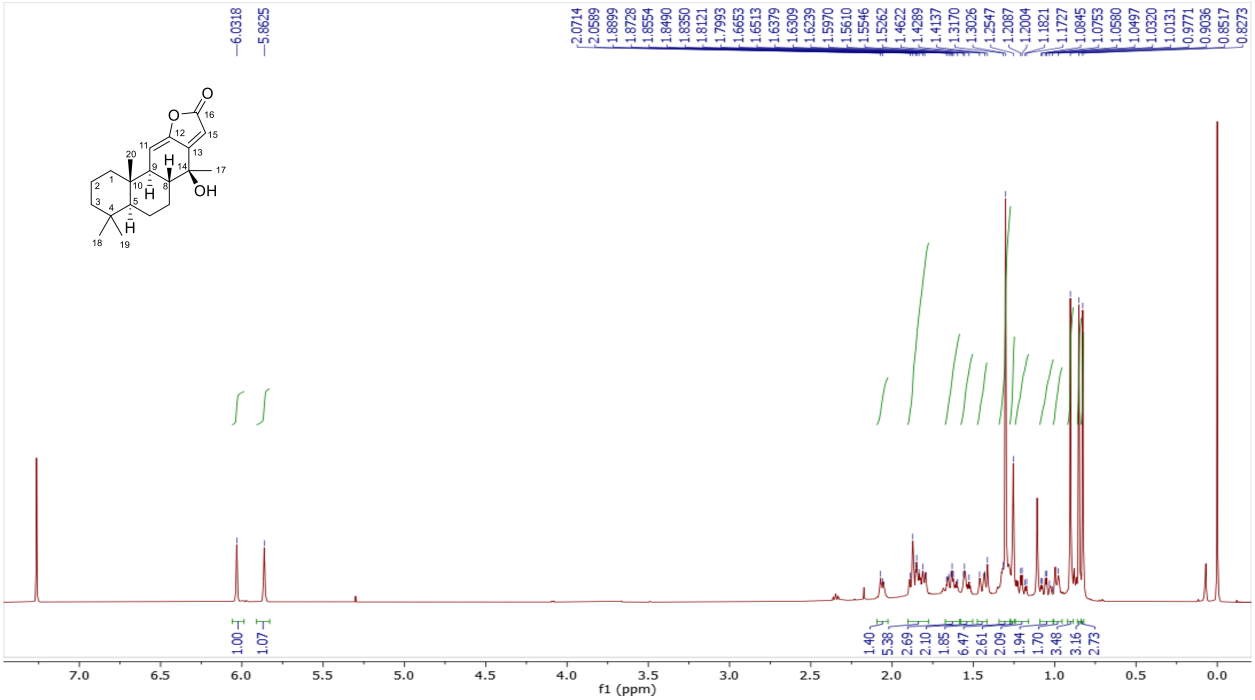

**Figure S1**. $^{1}$H NMR (500 MHz, $CDCl_3$) spectrum of 14β-hydroxycassa-11(12),13(15)-dien-12,16-olide (**1**).

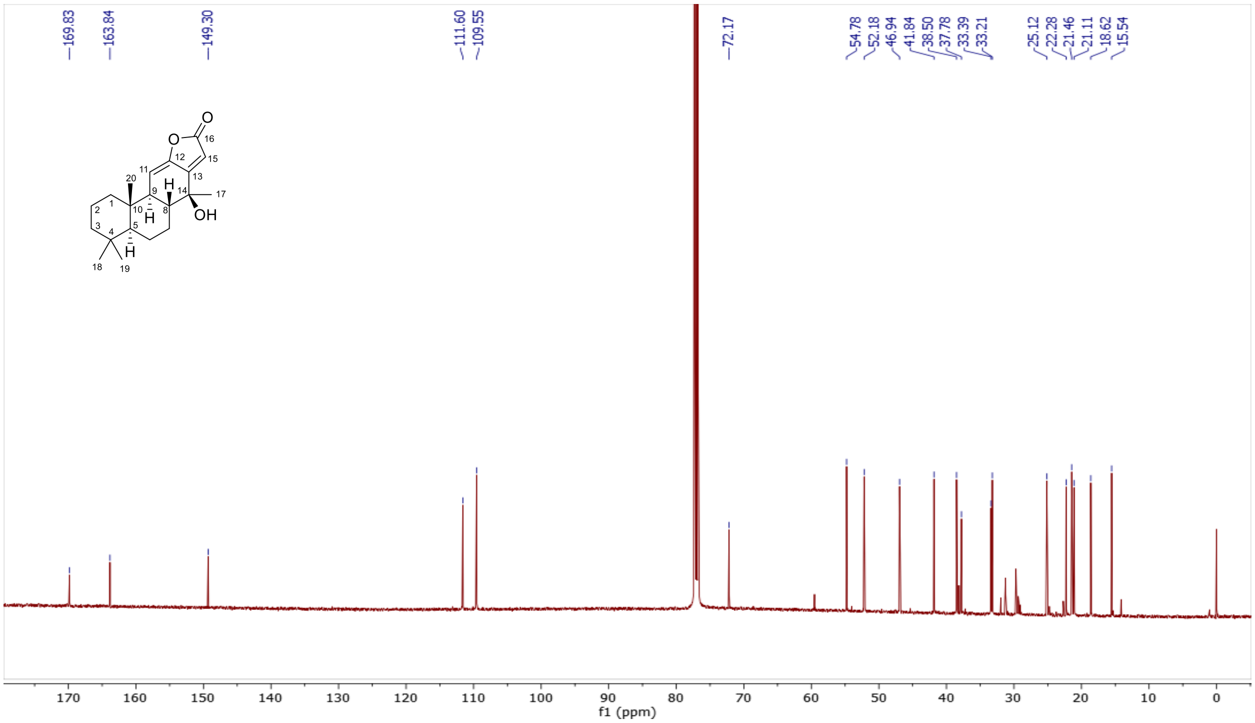

**Figure S2**. $^{13}$C NMR (125 MHz, $CDCl_3$) spectrum of 14β-hydroxycassa-11(12),13(15)-dien-12,16-olide (**1**).

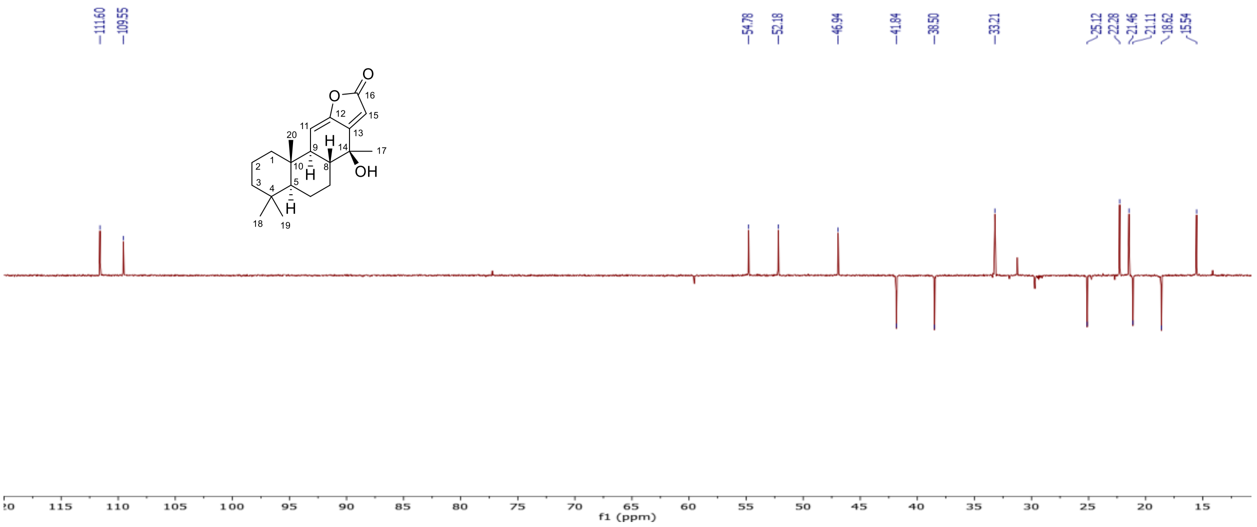

**Figure S3**. DEPT135 spectrum of 14β-hydroxycassa-11(12),13(15)-dien-12,16-olide (**1**).

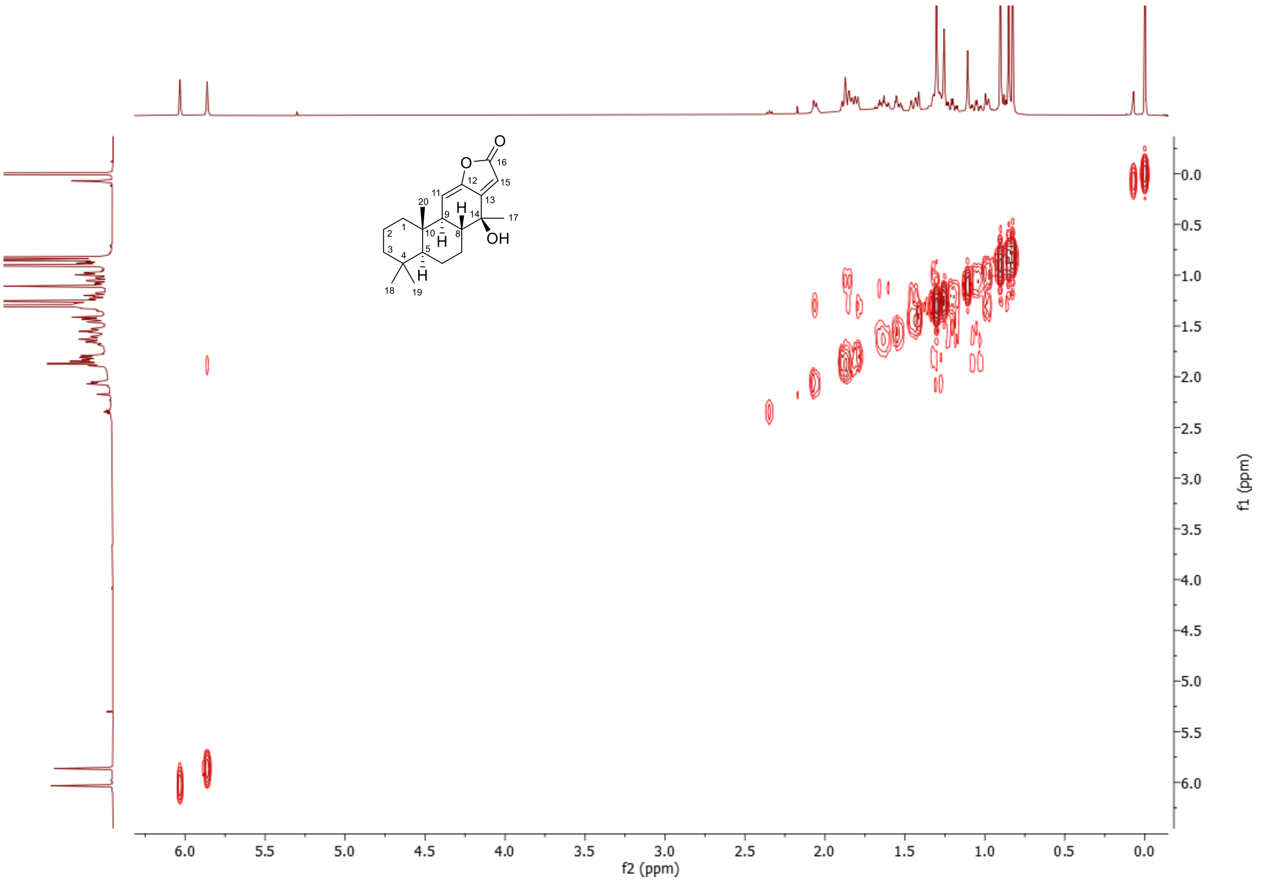

**Figure S4**. $^{1}H$-$^{1}H$ COSY spectrum of 14β-hydroxycassa-11(12),13(15)-dien-12,16-olide (**1**).

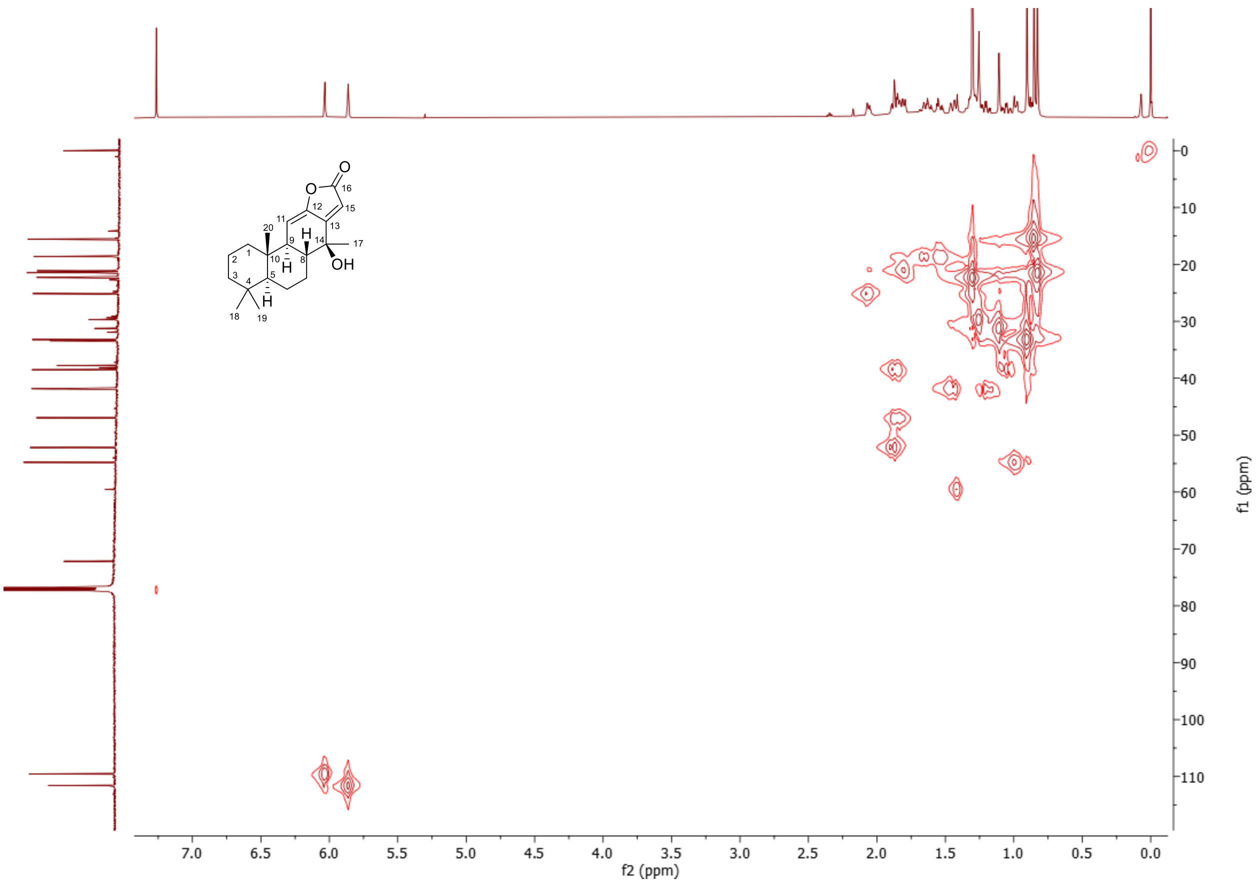


**Figure S5**. HMQC spectrum of 14β-hydroxycassa-11(12),13(15)-dien-12,16-olide (**1**).

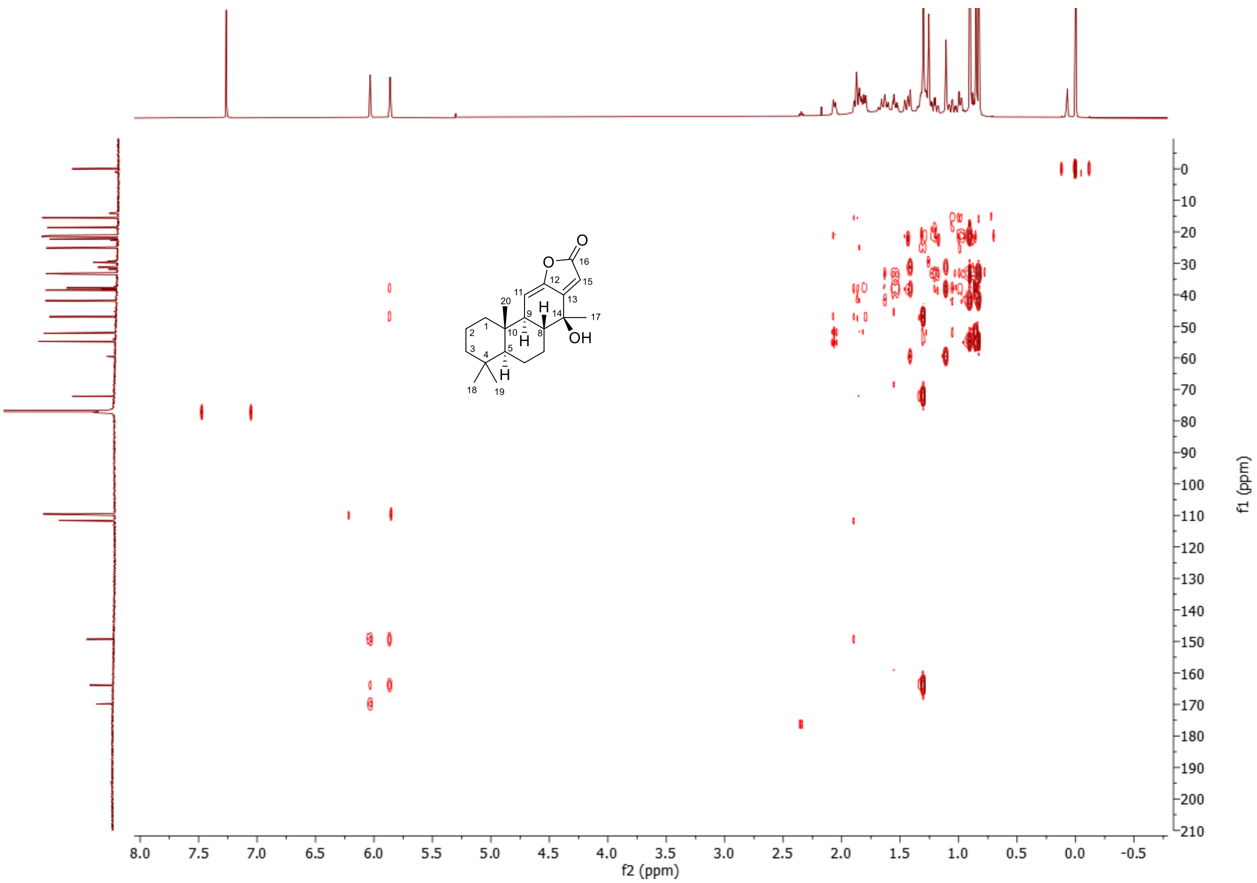


**Figure S6**. HMBC spectrum of 14β-hydroxycassa-11(12),13(15)-dien-12,16-olide (**1**).

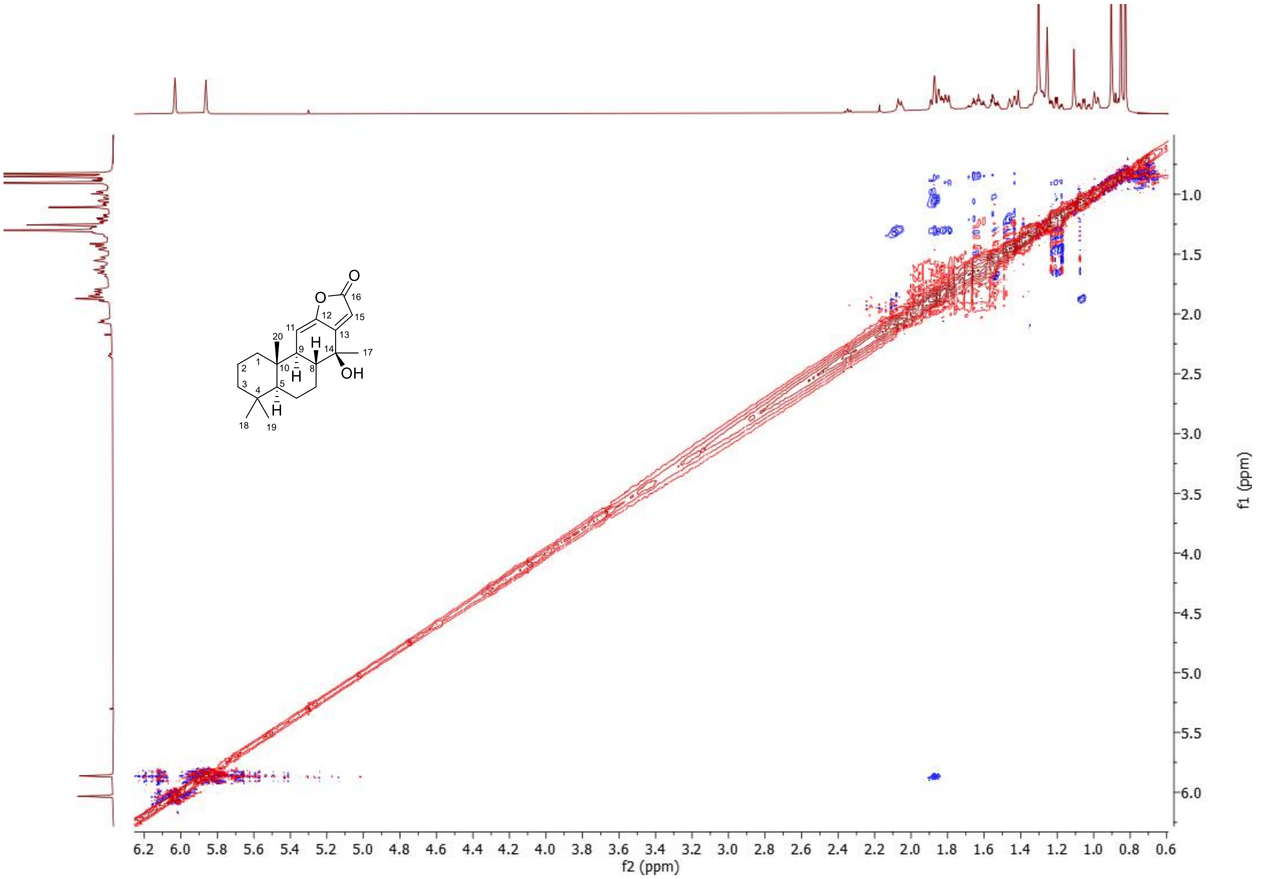


**Figure S7**. NOESY spectrum of 14β-hydroxycassa-11(12),13(15)-dien-12,16-olide (**1**).

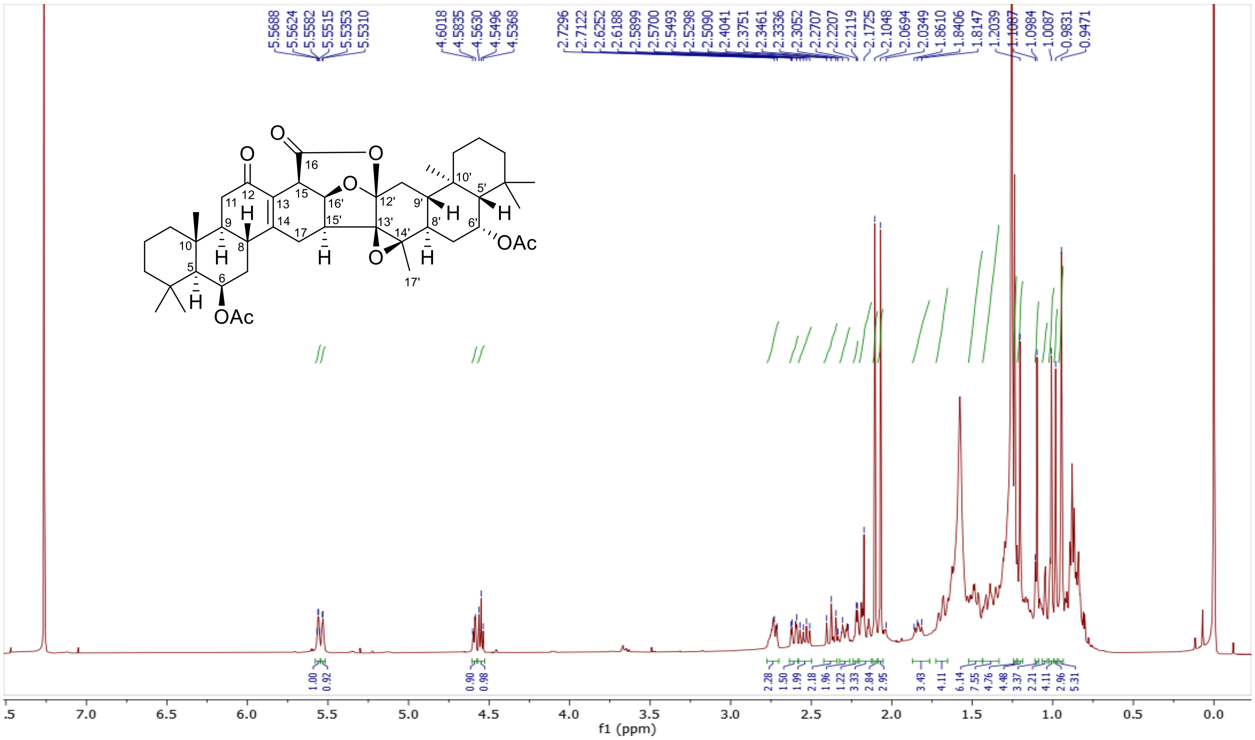

**Figure S8**. $^{1}H$ NMR (500 MHz, $CDCl_3$) spectrum of 6′-acetoxypterolobirin B (**3**).

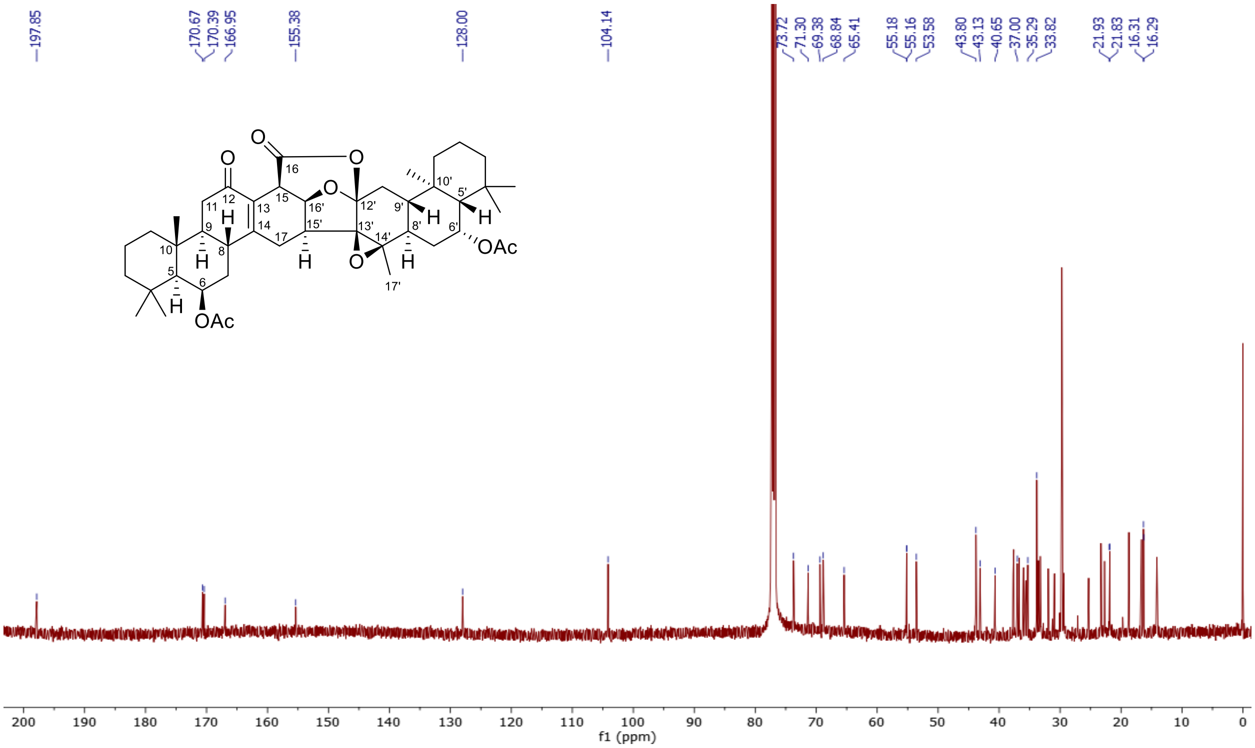

**Figure S9**. $^{13}C$ NMR (125 MHz, $CDCl_3$) spectrum of 6′-acetoxypterolobirin B (**3**).

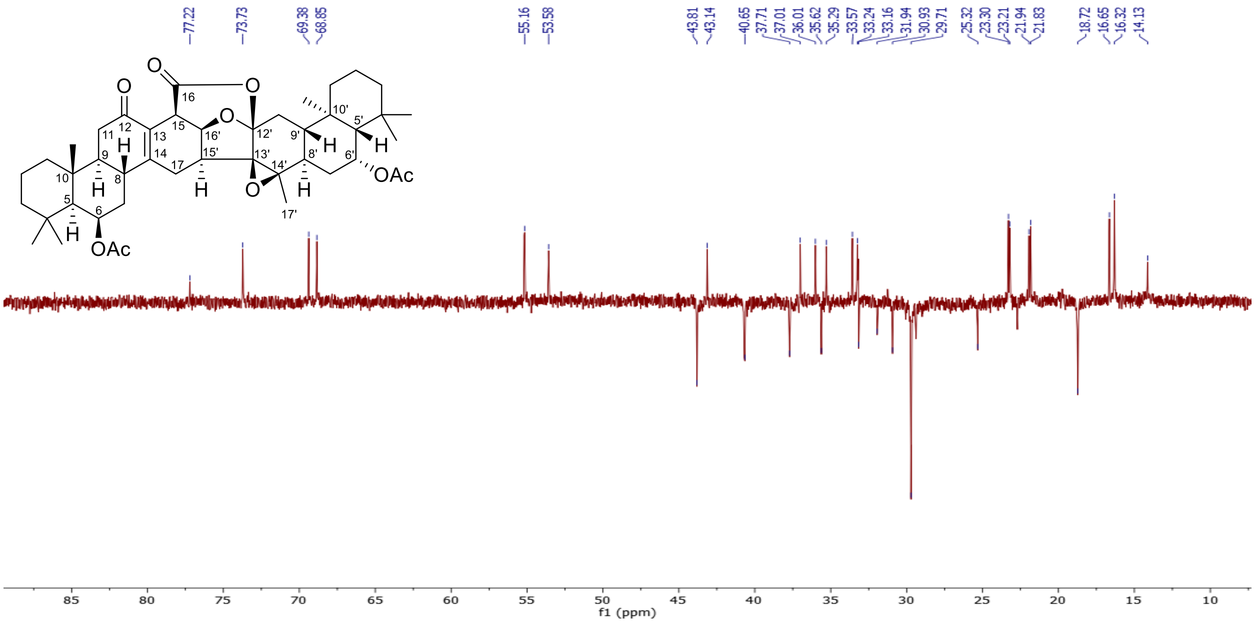

**Figure S10**. DEPT135 spectrum of 6′-acetoxypterolobirin B (**3**).

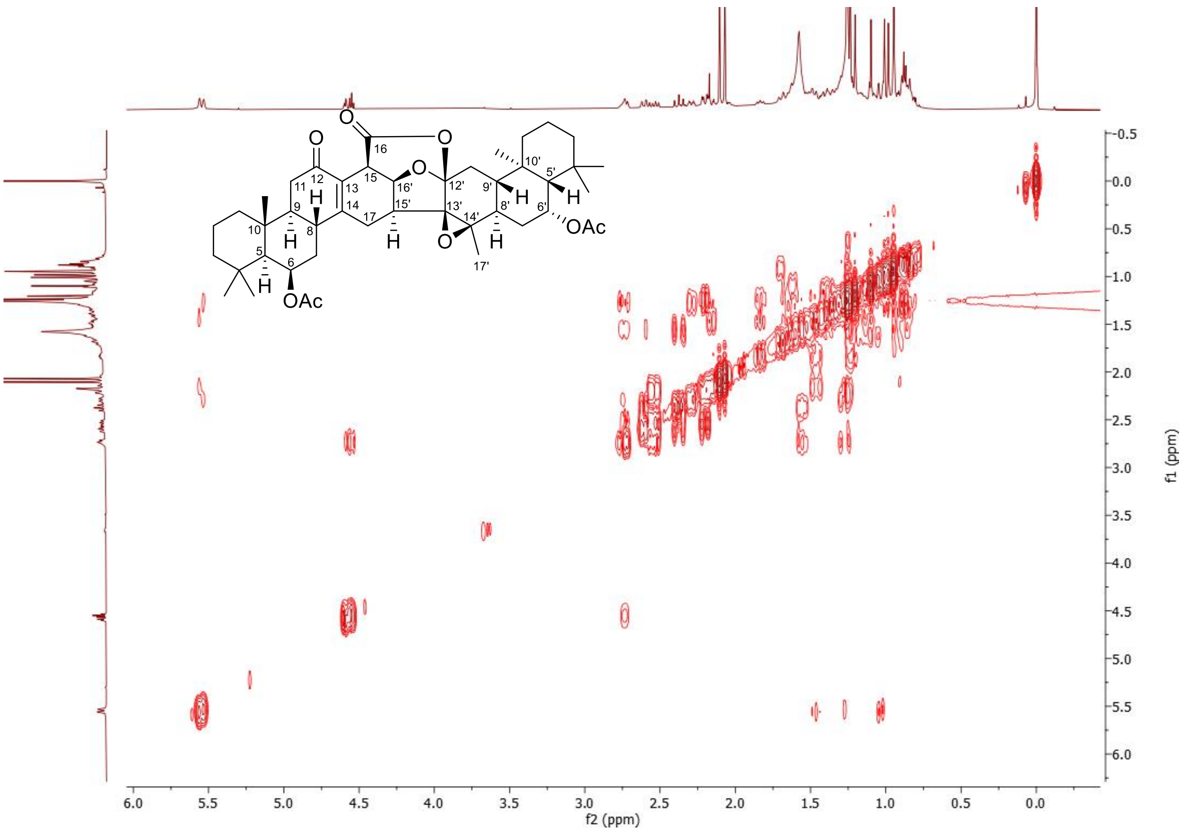

**Figure S11**. $^{1}$H,$^{1}$H COSY spectrum of 6′-acetoxypterolobirin B (**3**).

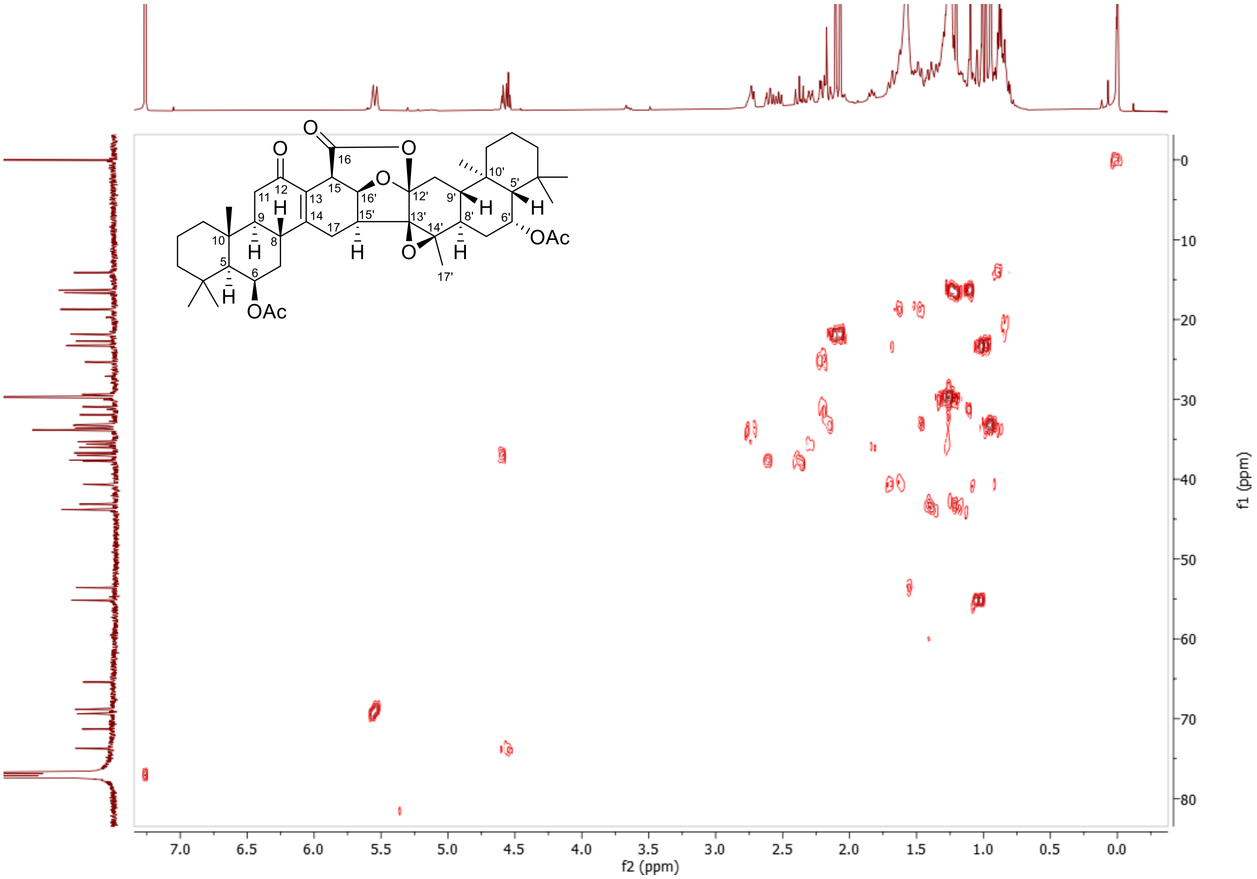


**Figure S12**. HMQC spectrum of 6′-acetoxypterolobirin B (**3**).

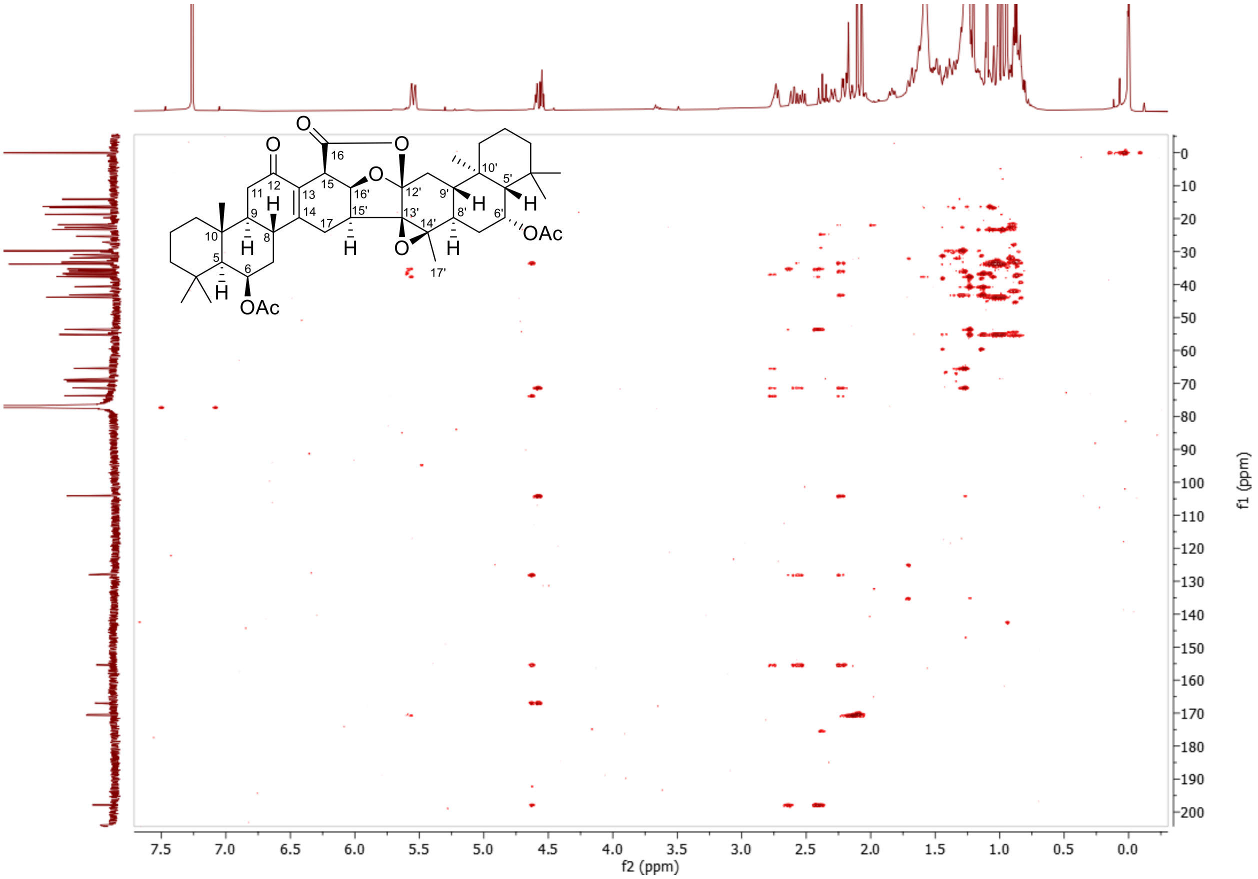


**Figure S13**. HMBC spectrum of 6′-acetoxypterolobirin B (**3**).

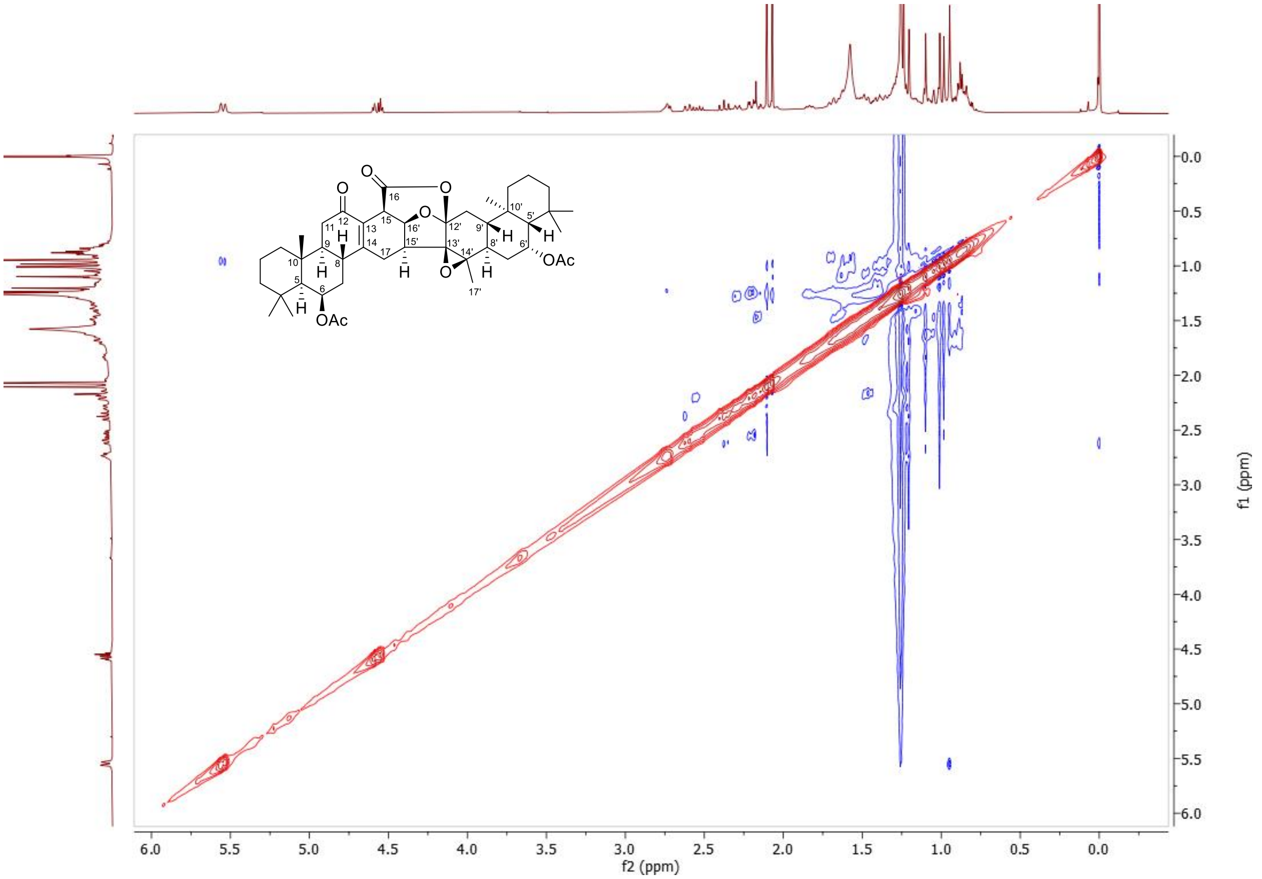


**Figure S14**. NOESY spectrum of 6′-acetoxypterolobirin B (**3**).

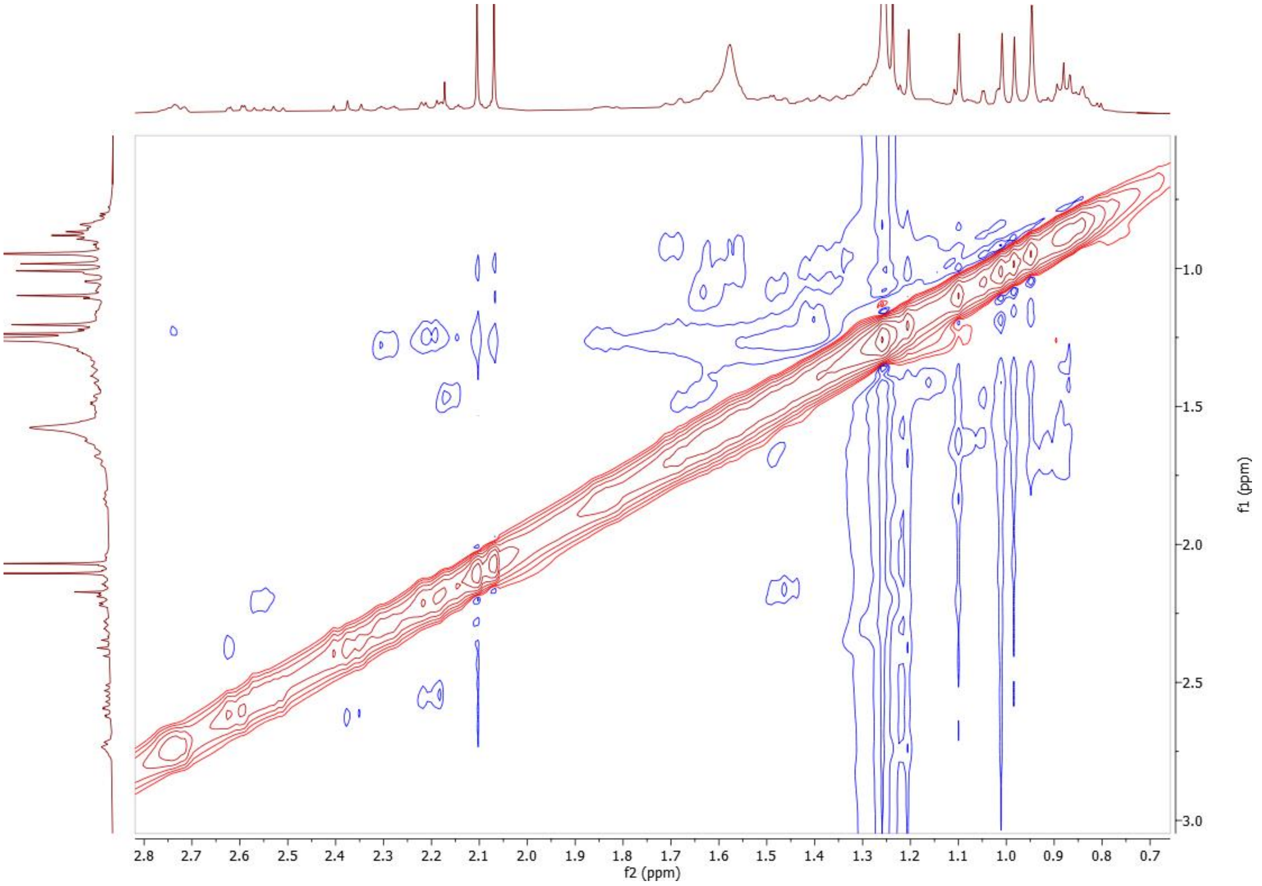


**Figure S15**. Magnified NOESY spectrum of 6′-acetoxypterolobirin B (**3**).
